# Effects of rIL2/anti-IL2 antibody complex on chikungunya virus-induced chronic arthritis in a mouse model

**DOI:** 10.1038/s41598-023-34578-x

**Published:** 2023-05-05

**Authors:** Sarah R. Tritsch, Abigail J. Porzucek, Arnold M. Schwartz, Abigale M. Proctor, Richard L. Amdur, Patricia S. Latham, Gary L. Simon, Christopher N. Mores, Aileen Y. Chang

**Affiliations:** 1 Milken Institute School of Public Health, The George Washington University, Washington, D.C., USA; 2 School of Medicine and Health Sciences, The George Washington University, Washington, D.C., USA

**Keywords:** Viral infection, Viral infection, Experimental models of disease

## Abstract

Chikungunya virus (CHIKV) is characterized by disabling joint pain that can cause persistent arthritis in approximately one-fourth of patients. Currently, no standard treatments are available for chronic CHIKV arthritis. Our preliminary data suggest that decreases in interleukin-2 (IL2) levels and regulatory T cell (Treg) function may play a role in CHIKV arthritis pathogenesis. Low-dose IL2-based therapies for autoimmune diseases have been shown to up-regulate Tregs, and complexing IL2 with anti-IL2 antibodies can prolong the half-life of IL2. A mouse model for post-CHIKV arthritis was used to test the effects of recombinant IL2 (rIL2), an anti-IL2 monoclonal antibody (mAb), and the complex on tarsal joint inflammation, peripheral IL2 levels, Tregs, CD4 + effector T cells (Teff), and histological disease scoring. The complex treatment resulted in the highest levels of IL2 and Tregs, but also increased Teffs, and therefore did not significantly reduce inflammation or disease scores. However, the antibody group, which had moderately increased levels of IL2 and activated Tregs, resulted in a decreased average disease score. These results suggest the rIL2/anti-IL2 complex stimulates both Tregs and Teffs in post-CHIKV arthritis, while the anti-IL2 mAb increases IL2 availability enough to shift the immune environment towards a tolerogenic one.

## Introduction

Chikungunya virus (CHIKV) is an alphavirus spread by the *Aedes aegypti* mosquito that is characterized by disabling joint pain that can cause persistent arthritis in approximately one-fourth of patients. For most CHIKV infections, acute symptoms typically resolve within 7 to 10 days; however, multiple studies have reported patients experiencing persistent joint pain months or years after the initial infection. In a study involving 485 CHIKV patients from Colombia, 25% reported persistent joint pain 20 months after infection, and 13% reported pain 40 months after infection^1,2^. The mouse model for post-CHIKV arthritis involves footpad inoculation of wild-type immunocompetent C57BL/6 mice, which causes localized swelling and systemic infection. In a pilot study involving the post-CHIKV arthritis mouse model, we determined that CHIKV footpad infection results in histologic evidence of arthritis, synovitis, periostitis, and myositis that persist to 21 days post-infection (dpi)^3^.

Currently, there are no standard treatments available for chronic CHIKV arthritis. Our preliminary data suggest that decreases in the cytokine interleukin-2 (IL2) during the acute stage of infection and the resulting alteration of regulatory T cell (Treg) function may play a role in CHIKV arthritis pathogenesis^3,4^. Novel low-dose IL2-based therapies for autoimmune diseases have been shown to up-regulate Tregs and may be of use in CHIKV arthritis flares in humans^5,6^. The short half-life of IL2 can be prolonged in vivo using various anti-IL2 monoclonal antibodies to form an IL2/anti-IL2 antibody complex^5,7^. One study found that IL2/anti-IL2 antibody complexes displayed an extended lifespan in vitro and in vivo, with the activity of IL2 alone tapering after 2 and 4 h, while the activity of the complex lasted greater than 24 h and 72 h, respectively^7^. Unlike other IL2 antibodies, the JES6-1 neutralizing antibody complexed with IL2 selectively induces Tregs expansion due to their high constitutive expression of the high-affinity IL2 receptor, IL2R∝ (CD25). The specificity stems from the JES6-1 antibody binding to IL2 in a way that sterically hinders the interaction between IL2 and the low-affinity receptors IL2Rβ (CD122) and IL2Rγ (CD132) found on other immune cells^7,8^.

We hypothesized that chronic CHIKV arthritis activity was associated with deficient IL2-mediated Treg levels; therefore, we tested the therapeutic effects of recombinant IL2 (rIL2), an anti-IL2 JES6-1 monoclonal antibody (mAb), and an rIL2/anti-IL2 JES6-1 mAb complex on post-CHIKV arthritis in our mouse model. Our aims were 1) to describe the role of IL2 in Treg expansion and corresponding arthritis severity in CHIKV arthritis and 2) to determine the role of IL2 therapy in treating CHIKV arthritis in a mouse model.

## Results

### Tarsal joint inflammation peaks at six days post-CHIKV infection and returns to baseline prior to treatment

**Figure 1:**
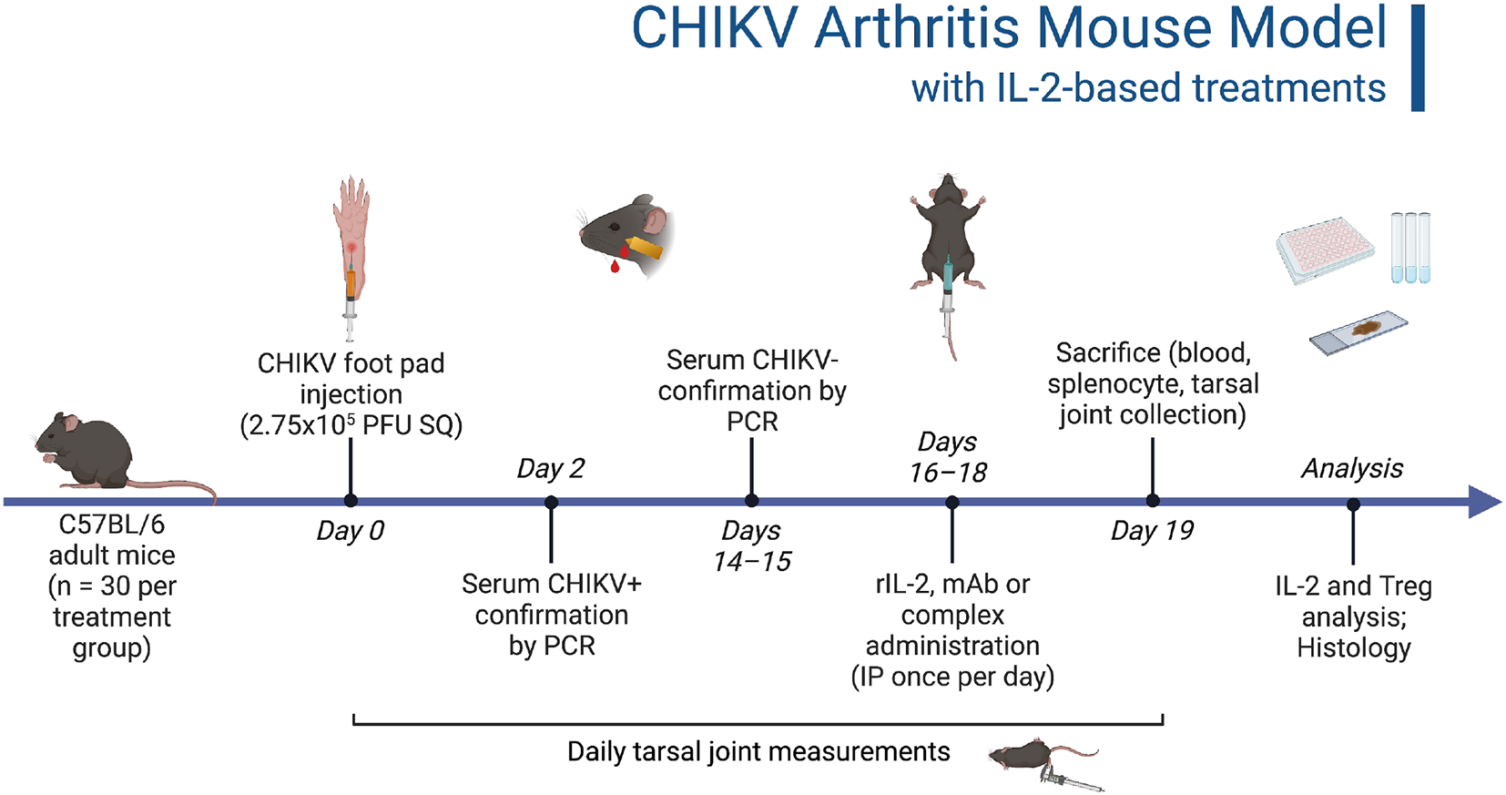
CHIKV post-acute arthritis mouse model with IL2-based treatments. C57BL/6 adult mice were infected with CHIKV via footpad injection. Mice were confirmed positive for CHIKV at two days post-infection (dpi) and confirmed negative by 16 dpi. Recombinant IL2, anti-IL2 monoclonal antibody (mAb), or a rIL2/anti-IL2 mAb complex were administered via intraperitoneal injection once per day for three days, starting at 16 dpi. Mice were sacrificed at 19 dpi, and spleen, hind limbs, and blood were collected for future analysis. Tarsal joints were measured daily.

Figure 1 illustrates the stepwise flow of the protocol. At two days post-infection (dpi), all mice were confirmed CHIKV-positive by qRT-PCR. By 14 dpi, most mice were CHIKV-negative, and by 16 dpi, all mice were confirmed CHIKV-negative. The raw data is included as a supplementary dataset and includes mouse gender, tarsal joint measurements, serum IL2 concentrations, flow cytometry populations, and histology scores.

Due to the negative side effects observed by a previous study that treated CHIKV-infected mice with a rIL2/anti-IL2 mAb complex using 50 μg of anti-IL2 mAb, the anti-IL2 mAb concentration was reduced in an attempt to minimize these side effects^9^. A literature search for anti-IL2 complexes in mice revealed multiple studies that used 5 μg of anti-IL2 in their complexes resulting in the successful expansion of Treg cells^10,11^. Thus, the anti-IL2 mAb per mouse was reduced to 5 μg when administered alone or in the complex. The 1.5 μg dose of recombinant mouse IL2 was kept the same as in previous studies^6,9^.

**Figure 2:**
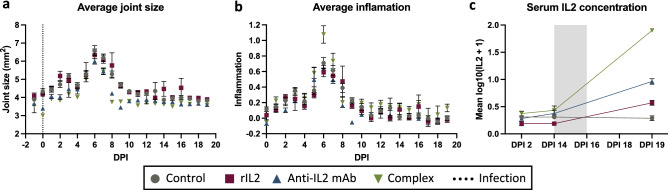
Tarsal joint measurements and inflammation are similar among treatment groups, while serum IL2 levels vary significantly. Mice were infected with CHIKV subcutaneously in the left hind foot near the tarsal joint. PBS, rIL2, anti-IL2 mAB or complex were administered to mice on days 16–18 post-CHIKV infection. Error bars represent SEM. (**a**) Average joint size of mice by treatment group on each day of the study, starting one day before infection (day -1), which served as the baseline joint size for calculating inflammation. The average joint size was calculated by multiplying the height by the breadth of the left tarsal joint. (**b**) Tarsal joint inflammation was calculated using [(x – day -1)/day -1)], where x is the footpad size measurement for a given day post-infection and day -1 is the day before infection. (**c**) Serum IL2 concentrations were measured on days 2, 14, and 19 after infection and were normalized using log10(IL2 + 1). The shaded region denotes the treatment period.

Tarsal joint measurements exhibited an overall increase in infected tarsal joints' swelling that peaked at six dpi and returned to baseline by ten dpi for all treatment groups. (Fig. 2a). During treatment (days 16–19), there was a trend level time effect (*p* = 0.07) with a reduction over time, on average. However, the interaction between the treatment group and day was insignificant (*p* = 0.41), indicating that the change over time was similar between treatment groups. Results were also similar using the inflammation measure, calculated as a change from baseline joint size divided by baseline joint size (Fig. 2b).

### Increased peripheral IL2 levels are associated with treatments

IL2 is essential for the proliferation, maintenance, and activation of Tregs^5^. Low doses of IL2 selectively expand and activate Tregs in multiple autoimmune and inflammatory diseases^6,10,12^. To determine the impact of IL2-based treatments on peripheral IL2 levels in the mice, serum IL2 analysis was performed. IL2 distributions were normalized using log10(IL2 + 1) for analysis. IL2 levels were low and stable in all treatment groups between 2 and 14 dpi (before treatment). However, at 19 dpi (after treatment), IL2 levels increased significantly compared to 14 dpi (before treatment) in the three experimental groups, with a 2.3-fold increase in rIL2-treated mice, a 6.9-fold increase in IL2 mAb-treated mice, and a 57.8-fold increase in complex-treated mice compared to the control group (Fig. 2c). IL2 levels in the control group did not change. In the mixed model examining change in IL2 levels from days 14 to 19 by treatment group, the main effects of the treatment group and time were both significant (both *p* < 0.0001), as was the interaction between treatment group and time (*p* < 0.0001).

### Complex treatment increases Treg and CD4 + Teff cell proliferation and activation

**Figure 3:**
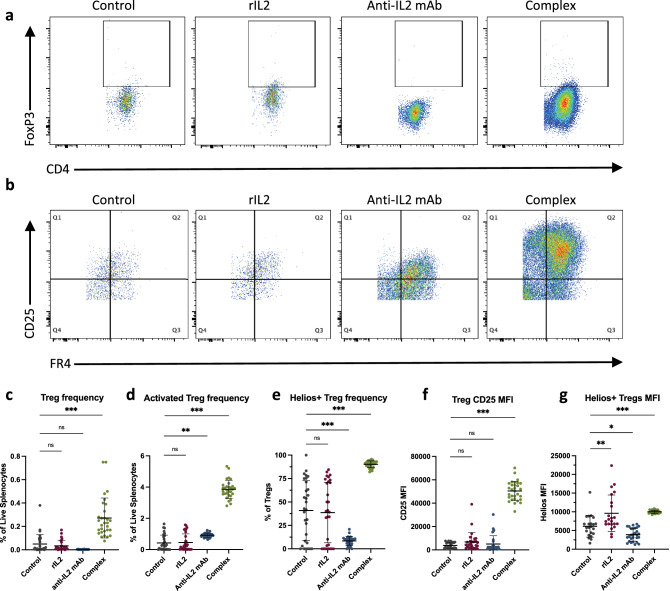
Therapeutic complex treatment significantly increases the frequency and activation of regulatory T cells. Mice were infected with CHIKV and treated with IL2-based treatments on days 16–18 post-infection, and splenocytes were collected and stained for flow cytometry on day 19 post-infection. (**a**) Representative scatterplots show CD4 and FoxP3 gated on CD4 + CD25 + live splenocytes. The box denotes the FoxP3 + populations. (**b**) Representative scatterplots show CD25 and FR4 gated on CD4 + CD25 + FR4 + cells. Q1 is the CD25^hi^FR4^lo^ activated Teff population, Q2 is the CD25^hi^FR4^hi^ activated Treg population, and Q4 is the CD25^lo^FR4^lo^ naïve T cell population. (**c**) CD4 + CD25^hi^FoxP3 + Treg cell frequency out of live splenocytes. (**d**) FR4^hi^CD25^hi^ activated Treg cell frequency out of live splenocytes. (**e**) Frequency of Helios-positive Tregs out of all Tregs. (**f**) CD25 median fluorescence intensity (MFI) on Tregs. (**g**) Helios MFI on Helios + Tregs. *Significant change from control (*p < 0.05, **p < 0.001, ***p < 0.0001).

Tregs are important immunosuppressors that express the high-affinity IL2 receptor alpha-chain (CD25), which is targeted by low-dose IL2 therapies^5,7^. The binding of IL2 to CD25 on Tregs initiates their expansion and activation^13^. Additionally, FR4 can be used with CD25 as a marker for FR4^hi^CD25^hi^ antigen-activated or clonally expanded Treg cells (activated Tregs)^14^. To understand the role of IL2 in Treg populations in CHIKV arthritis, flow cytometry was performed on mouse splenocytes (Table 1), which determined that Treg frequency and activation levels differed significantly across all treatment groups (p < 0.0001, Fig. 3). Representative scatterplots are depicted in Figs. 3a,b. There was a significant increase in the percent Tregs out of live splenocytes and CD25 median fluorescence intensity (MFI) in complex-treated mice compared to controls (*p* < 0.0001) and compared to rIL2 alone (*p* < 0.0001, Fig. 3c,f). When FR4 and CD25 were used to determine the level of activated Tregs, a slight increase in antibody-treated mice and a greater increase in complex-treated mice was observed compared to the control group (both p < 0.0001) and the rIL2 group (both *p* < 0.0001, Fig. 3d). There was also a significant increase in the Helios activation marker in complex-treated mice and a significant decrease in antibody-treated mice compared to the control group (both *p* < 0.0001) and the rIL2 group (both *p* < 0.0001, Fig. 3e,g).

**Table 1:** Regulatory and effector T cell levels by treatment group (mean ± SD) determined by flow cytometry on mouse splenocytes collected and stained at 19 dpi. One-way ANOVA *p* values are listed for comparisons between groups.

Population	Control	rIL2	Anti-IL2 mAb	Complex	*p* value
% Tregs out of live splenocytes	0.05 ± 0.08	0.03 ± 0.04	0.003 ± 0.00	0.27 ± 0.17***	< 0.0001
% Tregs out of CD4 + cells	0.26 ± 0.47	0.51 ± 1.00	0.02 ± 0.01	1.95 ± 1.24***	< 0.0001
% activated Tregs out of live splenocytes	0.42 ± 0.50	0.45 ± 0.55	0.92 ± 0.14**	3.86 ± 0.58***	< 0.0001
% Helios + Tregs	40.91 ± 31.78	38.58 ± 32.20	8.39 ± 4.81***	90.20 ± 3.68***	< 0.0001
Helios MFI	6128 ± 2905	9605 ± 4882**	3896 ± 1732*	10,003 ± 425***	< 0.0001
Treg CD25 MFI	3840 ± 2501	6860 ± 7855	5065 ± 7107	50,439 ± 8294***	< 0.0001
% Teffs out of live splenocytes	1.36 ± 0.73	1.56 ± 0.77	2.79 ± 0.38***	6.77 ± 0.91***	< 0.0001
Teff CD25 MFI	1272 ± 306.2	1465 ± 414.4	922.1 ± 82.26*	5673 ± 974.2***	< 0.0001
% activated Teffs out of live splenocytes	0.09 ± 0.08	0.10 ± 0.09	0.19 ± 0.06**	1.05 ± 0.17***	< 0.0001
% naïve T cells out of live splenocytes	0.31 ± 0.37	0.44 ± 0.48	0.45 ± 0.07	0.71 ± 0.11***	< 0.0001
Activated Treg: Teff ratio	3.60 ± 3.21	3.24 ± 2.56	5.00 ± 1.00*	3.70 ± 0.45	0.0099

*MFI* median fluorescence intensity.

Treg = CD4 + CD25^hi^FoxP3 + regulatory T cell, Teff = CD4 + CD25^hi^FoxP3 + effector T cell, activated Treg = FR4^hi^CD25^hi^, activated Teff = FR4^int^CD25^int/hi^, naïve T cell = FR4^lo^CD25^lo^.

*Significant change from control (*p < 0.05, **p < 0.001, ***p < 0.0001).

**Figure 4:**
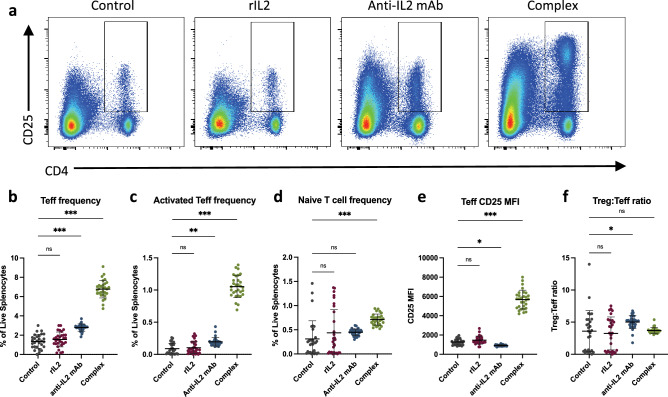
Anti-IL2 mAB and complex treatments significantly increase the frequency and activity of CD4 + effector T cells. Mice were infected with CHIKV and treated with IL2-based treatments on days 16–18 post-infection, and splenocytes were collected and stained for flow cytometry on day 19 post-infection. (**a**) Representative scatterplots show CD4 and CD25 gated on FoxP3- live splenocytes. The box denotes the CD4 + CD25 + FoxP3- populations. (**b**) Frequency of CD4 + CD25 + FoxP3- Teff cells out of live splenocytes. (**c**) FR4^int^CD25^int-^^hi^ activated Teff cell frequency out of live splenocytes. (**d**) FR4^lo^CD25^lo^ naïve T cell frequency out of live splenocytes. (**e**) CD25 median fluorescence intensity (MFI) on Teffs. (**f**) The ratio of activated Treg cells to activated Teff cells. *significant change from control (*p < 0.05, **p < 0.001, ***p < 0.0001).

Studies in CHIKV-infected *CD4*-knockout mice confirm that CD4 + effector T cells (Teff) are important drivers of CHIKV pathology^9,15^. In addition, activated Teff cells can express CD25, the alpha-chain of the high-affinity IL2 receptor that is usually a key marker for Tregs^16,17^. FR4 and CD25 can also be used to distinguish antigen-reactive CD4 + Teff cells (activated Teff, FR4^int^CD25^int-hi^) and unstimulated (naïve) T cells (FR4^lo^CD25^lo^)^14^. Flow cytometry of mouse splenocytes revealed that Teff frequency (Fig. 4a,b) increased significantly in both the complex-treated and the antibody-treated groups compared to the control (*p* < 0.0001) and rIL2 (*p* < 0.0001) groups. The CD25 MFI on the Teff cells decreased slightly with marginal significance in the antibody group (*p* < 0.05) and increased more than fourfold in the complex group (*p* < 0.0001) compared to the control group (Fig. 4d). Additionally, there was a slight increase in the frequency of activated Teff in the antibody group (*p* = 0.001) and a more considerable increase in the complex group (*p* < 0.0001) compared to the control group (Fig. 4c), as well as a significant rise in naïve T cells in the complex group compared to the control group (*p* < 0.0001, Fig. 4e). Finally, there was a moderate increase in the activated Treg to activated Teff ratio in the antibody group alone (*p* < 0.05, Fig. 4f). There were no significant changes in the frequency of Tregs, Helios-positive Tregs, Teff, or CD25 MFI of Teffs in the rIL2 group compared to the control group.

### Treatment with anti-IL2 antibody significantly decreases disease scoring in joint histology

Our previous study confirmed that CHIKV-infected mice exhibit signs of CHIKV joint pathology after the acute viral phase, and other studies have shown a connection between Treg levels and CHIKV arthritis^3,6^. The correlation between IL2-based treatments, Tregs, and CHIKV-associated chronic arthritis was further investigated using joint histology. For each mouse, two independent ratings were done for the individual and composite joint pathology scoring. Three methods were used to evaluate the test–retest reliability. First, the Pearson correlation coefficient (r) for reliability between ratings was examined with an *r* > 0.90 set as acceptable. The obtained *r* value was 0.904, indicating good reliability. Next, the Bland–Altman plot was reviewed to determine the fixed bias, which ideally should be zero. The fixed bias was calculated as 0.06, indicating good reliability. Finally, the weighted kappa was examined as a measure of inter-rater agreement. The weighted kappa was 0.78, indicating acceptable reliability (95% confidence interval 0.71–0.85).

**Figure 5:**
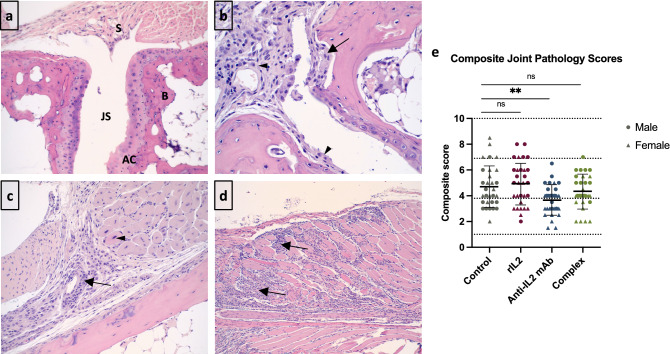
Histology images of mouse tarsal joints indicate disease severity. Mice were infected with CHIKV and treated with IL2-based treatments on days 16–18 post-infection, and tarsal joints were dissected 19 days post-infection. (**a**) Healthy tarsal joint from an uninfected mouse illustrating the synovium (S), joint space (JS), articular cartilage (AC), and subchondral bone (B)^3^; (**b**) CHIKV + mouse tarsal joint with bone and articular cartilage erosion (arrow), active synovitis (solid arrowhead), and pannus with articular cartilage erosion (open arrowhead); (**c**) CHIKV + mouse tarsal joint with mild myositis with degenerative myocytes (arrowhead), fasciitis (arrow), and periostitis with cortical bone erosion; and (**d**) CHIKV + mouse tarsal joint with severe myositis with degeneration and necrosis (arrows). (**e**) Composite joint pathology scores for each mouse by treatment group. Male mice are denoted by circles, and females by triangles. Scores for each mouse were calculated by adding the scores from each histological component. A score of 0 to < 4 was considered low severity, 4 to < 7 was moderate, and > 7 was severe, as illustrated by the horizontal dotted lines.

Figure 5 displays representative images of healthy (a) and CHIKV-infected (b–d) tarsal joints. Composite joint pathology scores of the left (infected) tarsal joints revealed a higher number of mice with severe arthritis in control (four mice) and rIL2 (six mice) groups compared to the antibody (zero mice) and complex (one mouse) groups (Fig. 5e). Additionally, after adjusting for sex, the mean composite joint pathology score was significantly lower in the antibody group than in any other group (*p* = 0.03). The antibody group was the only group with composite joint pathology scores significantly different from the control group (*p* = 0.0067). When looking at individual components of the histology, mice in all groups presented with synovitis and a majority had at least minor myositis and periosteal inflammation, while few mice showed cartilage or bone erosion (Supplemental Figure 1).

### Mouse gender affects peripheral IL2 levels and disease scores

The effects of mouse gender on the data were investigated. While males had larger joint sizes than females at baseline and over all time points, the sex effect on inflammation during treatment (days 16–19) was not significant (*p* = 0.14), indicating that overall inflammation was similar for male and female mice. The sex-by-day interaction was insignificant (*p* = 0.65), indicating that the change in inflammation during treatment did not differ by sex. Further, none of the Treg levels were significantly associated with sex, and controlling for sex did not affect the association of the treatment group with Treg levels.

However, in the IL2 model comparing day 14 (before treatment) to day 19 (after treatment), the 3-way interaction of the treatment group-by-time-by-sex was significant (*p* < 0.0001), indicating that the group-by-time interaction differed by sex. While the change in peripheral IL2 levels over time in control and rIL2 treatment groups was similar for males and females, females had a more considerable increase in IL2 over time in the antibody and complex treatment groups only. Additionally, the composite score was significantly higher for females after controlling for the treatment group (*p* < 0.0001), and the group effect remained significant after controlling for sex (*p* = 0.01). Proportions of mice with low, medium and high severity were 80%, 17%, and 3%, respectively, for males versus 42%, 43%, and 15% for female mice (*p* < 0.0001).

## Discussion

The investigation into the use of IL2-based therapeutics to treat post-CHIKV arthritis yielded interesting results. Daily tarsal joint measurements lacked significant changes between groups, which was expected based on our previous study^3^. IL2 analysis indicated stable levels of peripheral IL2 before treatment across all groups. This corresponds with the serum IL2 dynamics in the days following CHIKV infection in mice. Patil et al. described IL2 levels of less than 2 pg/ml in uninfected mice and increasing IL2 levels that peaked on day six post-infection before returning to baseline by day 17^18^. After treatment, there was a small but significant increase in IL2 concentrations in the rIL2 group, a larger increase in the antibody group, and the most substantial increase in the complex group. Interestingly, the antibody group was the only one with a significant decrease in the average composite joint pathology score. Conversely, flow cytometry analysis of splenocytes revealed that the complex was the only treatment to significantly increase the frequency and activity of FoxP3 + Tregs. When populations of activated Tregs defined as FR4^hi^CD25^hi^ were considered, there was a small but significant increase in the antibody group and a large increase in the complex group. There was a significant increase in the Teff cells in both the antibody and complex groups, but a decrease in Teff cell CD25 expression in the antibody group and an increase in the complex group, which is a marker of Teff activation. Consequently, the antibody group was the only group with a higher activated Treg to activated Teff cell ratio, pointing to a more tolerogenic immune environment compared to the other groups. Finally, sex analysis of the data revealed that females had worse histology outcomes corresponding to human disease; however, females in the antibody and complex groups were more responsive to treatment than males when considering peripheral IL2. This could be due to differences in size since male mice are typically larger than female mice.

**Figure 6:**
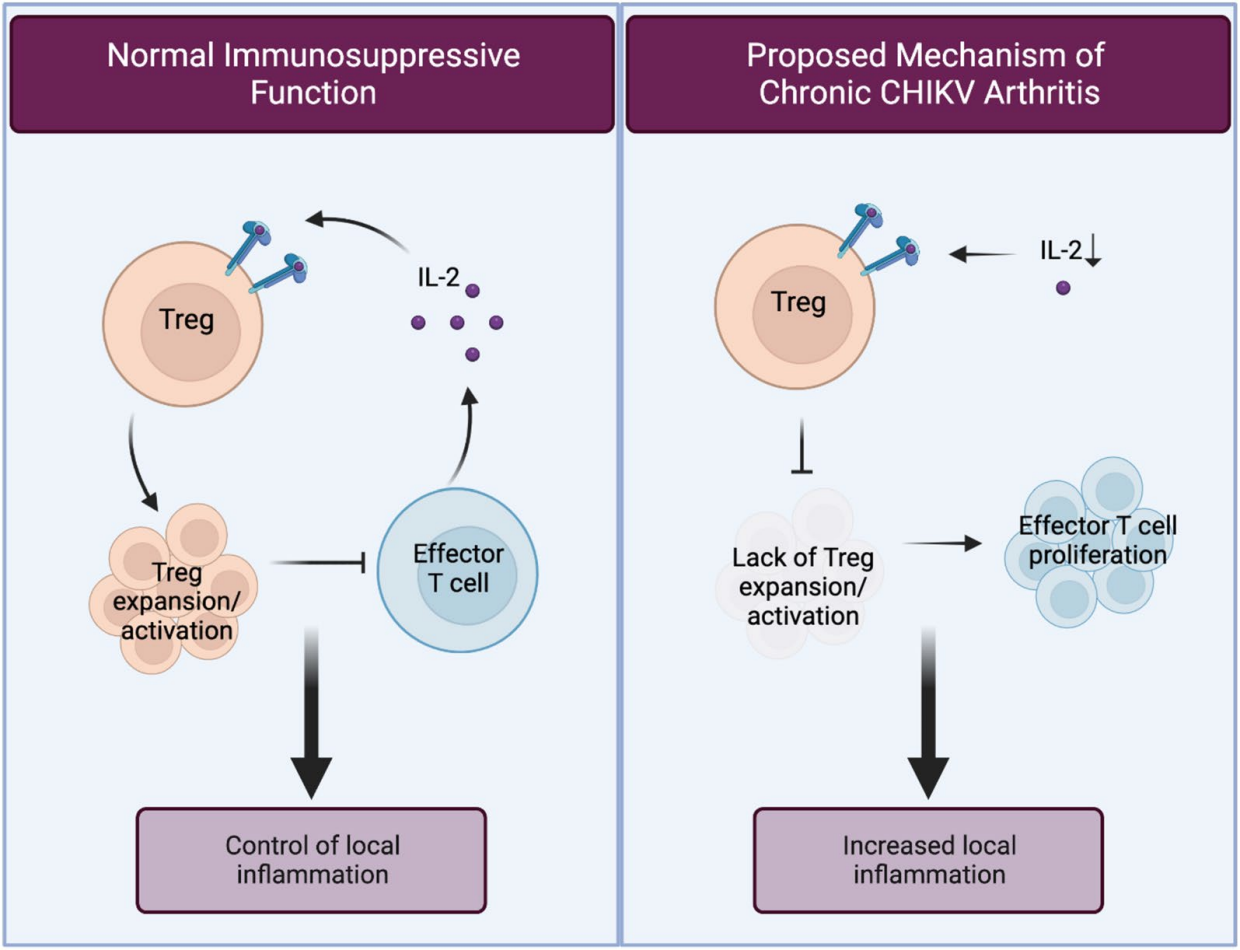
IL2 and T-reg immunology in normal immune function and chronic CHIKV arthritis.

Figure 6 depicts IL2 and T-reg immunology in normally functioning immune suppression and chronic CHIKV arthritis. The left panel illustrates the interaction of normal peripheral levels of IL2 and Tregs during normal suppressive function. In homeostasis, IL2 binds to the high-affinity receptor (CD25) on Tregs, which drives their expansion and activation. Tregs can then suppress the growth of effector T cells, thereby controlling inflammation. As shown in the right panel, deficient IL2 levels in CHIKV arthritis lead to a lack of Treg expansion and activation, allowing T effector cells to expand unregulated and leading to increased local inflammation^4,15^.

We hypothesized that IL2-based treatments such as recombinant IL2, an anti-IL2 mAb, or the two in complex would increase peripheral levels of IL2, therefore increasing the proliferation and activation of Tregs and depressing Teff cells. While there was not an expansion of Tregs in the antibody-treated group, there was an increase in Treg activation with only a slight increase in Teff frequency and a decrease in Teff activation, which corresponded with a significant decrease in disease severity in the tarsal joint. One reason for the low levels of Tregs in the periphery of antibody-treated mice may be the relocalization of Tregs to the site of tissue damage, as reported by Burzyn et al. in muscle damage^19,20^. Conversely, rIL2 alone did not expand or activate Tregs, allowing Teff cells to proliferate, while the complex treatment increased both effector and regulatory T cells.

A correlation between peripheral IL2 and Tregs was not observed in our study. This could be because IL2 metabolizes and is active in the lymph nodes rather than the periphery. Indeed, there is clinical trial data showing that when low-dose exogenous IL2 is administered to patients with autoimmune diseases on days 1–5, plasma Treg levels are augmented by day 8 with no significant increase in IL2 levels^12^. Furthermore, when maintenance dosing was administered every two weeks, Treg levels remained elevated at six months^12^. Therefore, short-term exogenous IL2 therapy may have a plausible effect on Treg levels.

Previous studies by Lee et al. (2015 and 2016) have investigated the effects of the rIL2/anti-IL2 complex administered before and at the peak of CHIKV infection in mice. Prophylactic treatment with the complex prevented arthralgia development, while treatment during acute infection caused infiltration of pathogenic CD4 + CD25 + Teff cells and intensified joint inflammation^6,9^. Our study aimed to determine the complex’s effects on active arthralgia after viral clearance, as this is when patients usually present for treatment of post-CHIKV arthritis. However, treatments had to be administered immediately after the virus was cleared since, unlike in humans, diseased joint pathology in mice will eventually clear on its own. Based on the results from Lee et al., it is possible that pathogen-activated Teff cells, which express high levels of CD25, were still in circulation in our mice, even after the virus was cleared. Indeed, Teff populations in the complex group had significantly higher CD25 expression, while the antibody group Teff populations had significantly lower CD25 expression. Additionally, the frequency of activated (antigen-reactive) Teff cells defined as FR4^int^CD25^int/hi^ moderately increased in the antibody group, with a tenfold increase in the complex group.

The histologic examination of the lower limb provided insight into the nature and progression of the inflammatory process. Encouragingly, none of the treatments worsened the disease outcome. Nonetheless, all treatment groups had mice with low, moderate, and severe disease scores. This is to be expected, as variation in disease progression after infection can occur among individual mice. Histologic scoring also demonstrated that synovitis was an early and prominent process, given that no case (control or therapeutic) was free of synovitis. Moreover, when synovitis was mild (individual histologic score of 1), there was no case of overall composite joint pathology severity (overall histologic score of 7, 8, 9, or 10). Similarly, when the synovitis score was marked (individual histologic score of 2), three-quarters of the cases had overall histologic scores of five or greater, indicative of moderate to severe disease. Further, most mice had at least mild myositis and periosteal inflammation. Few mice, however, showed signs of cartilage or bone erosion. This suggests that the disease pathology in mice begins with synovitis, progressing to muscle and periosteal involvement, and finally, cartilage and bone erosion in cases of severe disease. The variability of disease expression indicates that disease progression and host response pathways for injury and inflammatory reactivity differ in their extent among the cohort of cases.

Combined with previous studies, the data suggest that complexing rIL2 with the anti-IL2 mAb makes it more biologically available and, thus, has a more significant impact on the IL2-mediated increase in Tregs, which decreases overall inflammation and joint degradation^5,7^. However, there seems to be a goldilocks effect associated with these treatments, in which low-dose rIL2 alone is insufficient to drive a robust Treg response, while the complex increases IL2 levels too much, affecting the frequency and activity of Teff cells in addition to Tregs. This could be because the antibody concentration was not high enough to bind all the available exogenous rIL2 in the complex or because it allowed endogenous IL2 the opportunity to bind low-affinity receptors on Teff cells in complex-treated mice. The IL2 antibody, on the other hand, increased IL2 levels just enough to act on Treg cells but not Teff cells. We hypothesize that since the JES6-1 anti-IL2 antibody recognizes the IL2Rβγ (CD122) site of IL2, administration of the antibody alone binds available endogenous IL2, extending its half-life and ensuring its delivery to Treg cells^21^. Conversely, when JES6-1 is complexed with rIL2, endogenous (or excess exogenous) IL2 is free to bind low-affinity receptors on Teff cells, driving their proliferation and activation. A dose–response study in this mouse model could elucidate the anti-IL2 antibody mechanism for decreasing post-acute disease. Measuring the anti-IL2 antibody binding in the complex treatment could also help answer questions about its mechanism.

The non-target effect of an uncontrolled CD4 + T effector cell response in complex-treated mice requires further investigation since these cells are known drivers of CHIKV pathology^9,15^. This study used a post-acute mouse model, which requires IL2-based treatments to be administered immediately following viral clearance. Because the virus was only recently cleared from the mice, immune cells, such as the CD4 + T effector cells, may not have had time to clear the system prior to the administration of IL2 treatments. In humans, the post-acute complications are discovered months to years after the virus is cleared from the body and presents in a relapsing–remitting pattern^1,2^. Thus, it is probable that, unlike in the mice, patients suffering from chronic arthritis no longer have high levels of the IL2-sensitive CD4 + T effector cells. However, future studies should examine the effects of the rIL2/IL2 antibody complex on non-regulatory T cells in humans. Further, the co-administration of the complex with a Teff cell inhibitor, such as rapamycin, could dampen the nontarget effects of the primary treatment^13^.

A few limitations were noted during this study. First, treatments were not weight-based, which could account for the differences in response to therapies between male and female mice. Second, quantifying inflammation in the tarsal joints of infected mice is inherently difficult and led to small but perceptible variations in the joint size and inflammation data. Standard errors of the means (SEM) were included as error bars in the graphs to account for these variabilities. Additionally, this study used a post-acute mouse model, which differs from the clinical progression of post-acute human disease. Ultimately, these highly impactful findings indicate the need for human in vitro and clinical studies evaluating anti-IL2 monoclonal antibodies and the rIL2/anti-IL2 complex to treat CHIKV-related chronic arthritis.

## Methods

### Ethics statement

All animal care and procedures performed in this study were reviewed and approved by the George Washington University (GWU) Institutional Animal Care and Use Committee (IACUC), protocol #A456, which complies with the guidelines stated in the National Institutes of Health’s (NIH) Guide for the Care and Use of Laboratory Animals. This manuscript was written in accordance with the ARRIVE guidelines (Animal Research: Reporting of In Vivo Experiments).

### Mice

A total of 120 mice were included in this study. Equal numbers of 10-week-old male and female C57BL/6 J mice were obtained from The Jackson Laboratory (https://www.jax.org/). Mice were housed in groups of five in micro-isolator cages in the ABSL-3 laboratory at GWU at a constant room temperature (68–75°F) and humidity (30–70%) and with a 12-h light/dark cycle. Cages were lined with 1/8 inch corncob bedding, and each cage contained nesting materials and paperboard tunnels. Mice were given free access to food (PicoLab Verified 75 IF Irradiated) and water.

Mice were anesthetized with isoflurane, then injected with 25 µl (2.75 × 10^5^ plaque forming units (PFU)) of CHIKV subcutaneously in the caudoventral aspect of the left hind foot near the tarsal joint. Mice were monitored at least once daily for signs of illness, such as lethargy, joint swelling, lameness, and limb gnawing. At 19 dpi, all mice were euthanized via CO_2_ asphyxiation, followed by a cardiac stick.

### Virus

The strain of CHIKV used for this study was from the East, Central, and South African (ECSA) lineage that was initially isolated from a human case in South Africa (SAH-2123) and was supplied by the World Reference Center for Emerging Viruses and Arboviruses (Galveston, TX, USA). This strain was used in a previous study performed by our lab to determine a mouse model for studying chronic CHIKV arthritis^3^.

All studies performed with viable CHIKV were performed in Biosafety Cabinets in a certified BSL-3 laboratory and were conducted with the approval of the GWU Institutional Biosafety Committee, protocol #IBC-19–026.

CHIKV was passaged once in Vero cells (CCL-81, ATCC) in DMEM containing antibiotics and antimycotics, then quantified by viral plaque assay and quantitative real-time – polymerase chain reaction (qRT-PCR). The calculated titer of the stock virus was 1.1E7 PFU/ml, yielding a predicted limit of detection for the qRT-PCR assay of 0.01 PFU/ml.

### Viremia detection

At 48 h post-infection, 200 µl of blood was collected from the submandibular vein for serum analysis to confirm mice were CHIKV positive by qRT-PCR. The Zymo Research Quick DNA/RNA Kit was used to isolate viral RNA, and the viral load was quantified using the Invitrogen Superscript III Platinum One-Step qRT-PCR Kit and CHIKV primer/probe set^22^. RT-PCR was analyzed using a LightCycler 96 Instrument (Roche Diagnostics) with thermal cycling conditions as follows: one cycle at 50 °C for 900 s, one cycle at 95 °C for 120 s, and 45 cycles at 95 °C for 3 s, followed by 55 °C for 30 s and a cooling cycle at 37 °C for 30 s.

### Treatment with rIL2 and monoclonal antibody

At 16 dpi (the point at which all mice were CHIKV-negative), mice were treated once per day for three days by intraperitoneal injection with phosphate buffered saline (PBS) as a control, 1.5 µg/day recombinant mouse IL2, 5 µg/day anti-IL2 monoclonal antibody (mAb; clone JES6-1), or an rIL2/anti-IL2 complex containing 1.5 µg of rIL2 and 5 µg IL2 mAb. Thirty mice were included in each treatment group (15 males and 15 females).

### Serum IL2 analysis

Peripheral blood was collected from the submandibular vein at 2, 14, 16, and 18 dpi and via cardiac puncture at 19 dpi. Blood was allowed to clot at room temperature for 30 min, then centrifuged at 3000 × g for 10 min. Serum was aliquoted and stored at − 80 °C until analysis. The IL2 Mouse ProQuantum Immunoassay Kit (Invitrogen) was used to analyze serum IL2 levels.

### Flow cytometry

Spleens were dissected from mice following euthanasia, and splenocytes were collected by mashing the spleens through a 70-µm cell strainer. Red blood cells were lysed using ACK lysis buffer (Thermo Fisher Scientific). Cells were then stained for flow cytometry using the Biolegend antibodies FITC CD4 (clone RM4-5), PE-Cy7 CD25 (clone PC61.5), PE FoxP3 (clone MF-14), APC Helios (clone 22F6), and PerCP-Cy5.5 FR4 (clone 12A5), and Fixable Live/Dead Aqua stain from Invitrogen, as well as the FoxP3 Fixation/Permeabilization kit from eBiosciences. All antibodies were titrated prior to use in the study.

A BD LSRII flow cytometer was used to process samples, and analysis was performed using FlowJo Software. Compensation was performed for each fluorophore using CompOne beads (Thermo Fisher Scientific) and the ArC Amine Reactive Compensation Bead Kit (Thermo Fisher Scientific) for the Live/Dead stain. Treg cells were defined as CD4 + CD25^hi^FoxP3 + , and Teff cells were defined as CD4 + CD25 + FoxP3-. Further, FR4 (folate receptor 4) was used in combination with CD25 staining to define activated Tregs (FR4^hi^CD25^hi^), activated effector T cells (FR4^int^CD25^int-hi^), and naïve T cells (FR4^lo^CD25^lo^)^14^.

### Histology staining

After euthanasia, hindlimbs were dissected and fixed in 10% formaldehyde at 4 °C for a minimum of 48 h. Fixed tissues were rinsed with distilled water, and any remaining skin was removed. Decalcification of bones was achieved using a solution containing EDTA, sodium hydroxide, and polyvinylpyrrolidone (PVP) with a pH of 7.0–7.4. Tissues were submerged in the decalcification solution at 4 °C with agitation for 28 days, and the solution was changed every other day to maintain a pH of 7.0–7.4.

After decalcification, tissues were rinsed with distilled water and processed in PBS three times for 5 min each, in distilled water three times for 5 min each, in 50% ethanol for 5 min, and finally, in 70% ethanol for 5 min. After the final wash, the tissues were stored in 70% ethanol at 4 °C until processed.

To process for histopathological evaluation, tissue samples were dehydrated in increasing ethanol concentrations, xylene, and embedded in paraffin wax, from which five µm-thick sections were adhered to positively charged glass slides. Samples were stained with hematoxylin and eosin (H&E) for evaluation, and Masson’s trichrome stains were performed on select slides. All histologic evaluations were performed by a board-certified pathologist, who was blinded to the treatment group. Two blinded reads were performed one month apart, and the histologic score was averaged. All slides were evaluated using an Olympus BK46 microscope, and digital images were obtained using a Spot Imaging digital camera and Spot Pathsuite 2.0.

### Histologic inflammation scoring system

The generation of the semi-quantitative histologic inflammation scoring system used in this study has been previously described^3^. In short, a microscopic evaluation of bone and joint tissue assessed inflammatory reaction and injury in (1) the synovium, (2) articular cartilage, (3) skeletal muscle and soft tissue, (4) periosteum, and (5) cortical bone. The scoring ranged from zero (no injury/inflammation) to two (significant injury/inflammation) for each of the five components, with a total possible composite score of 0 to 10. The scoring system was based on the maximal degree of inflammation and injury seen on day seven in our pilot study^3^.

### Tarsal joint measurement

Tarsal joint width and breadth were measured daily using calipers and recorded in mm. Each joint measurement was performed three consecutive times and averaged to account for variability. The width was measured directly posterior to the tarsal joint foot pad projection, with only enough pressure to cause slight flaring of the toes. The breadth was measured at the same spot as the width, but the calipers were rotated around the foot 90 degrees. Footpad size was calculated as width x breadth, and the degree of inflammation was expressed as the increase in size relative to the pre-infection measurement (day -1), obtained by the following formula: [(x – day -1)/day -1)], where x is the footpad size measurement for a given day post-infection.

### Statistical analysis

SASS and GraphPad Prism Software (version 9.4.1) were used to analyze and graph data, with a *p* < 0.05 considered statistically significant. The change in joint inflammation was examined by treatment group, from baseline to the treatment period following CHIKV infection (baseline vs. days 16–19), using a random effects mixed model. This nests observations within subjects to account for correlated measurements on the same subjects over time. The ‘treatment’ time point was averaged across days 16–19. The treatment group-by-time interaction was also assessed to determine whether the slopes over time differed by group. The group and time main effects were tested to ascertain whether joint size differed across groups and time. Additionally, all analyses comparing groups were performed using a one-way ANOVA followed by Dunnett’s post-test comparing each treatment group to the control (PBS). Treg/Teff ratios were calculated, and mean ratios were analyzed by treatment group using a general linear model adjusted for sex. Histology composite scores were analyzed using a two-way ANOVA (mixed methods). Two independent ratings were done for each subject for the histology score, and test–retest reliability was assessed using the Pearson r, Bland–Altman plot and weighted kappa.

To determine sample size, raw data was used from Miner et al.’s seven-day change in foot-pad swelling in mice inoculated with CHIKV, comparing controls vs. mice treated with abatacept, to establish the expected effect size for IL2 treatment on ankle swelling^23^. Their effect size was 0.97. In order to have a power > 0.95 to detect this effect in a two-tailed (between-groups) *t* test with an alpha of 0.05, our study needed at least 29 mice per treatment group.

## Supplementary Information

Supplementary Figure 1.

Supplementary Information 2.

## Data Availability

The data generated and analyzed during the current study are available as a supplemental dataset.
