# Supplementary material for: Effects of rIL2/anti-IL2 antibody complex on chikungunya virus-induced chronic arthritis in a mouse model
Source: Sci Rep. 2023 May 5;13:7307. doi: 10.1038/s41598-023-34578-x (PMC10163023; doi:10.1038/s41598-023-34578-x)
Supplement: Supplementary file 1 — Supplementary Figure 1. [file 41598_2023_34578_MOESM1_ESM.pdf]

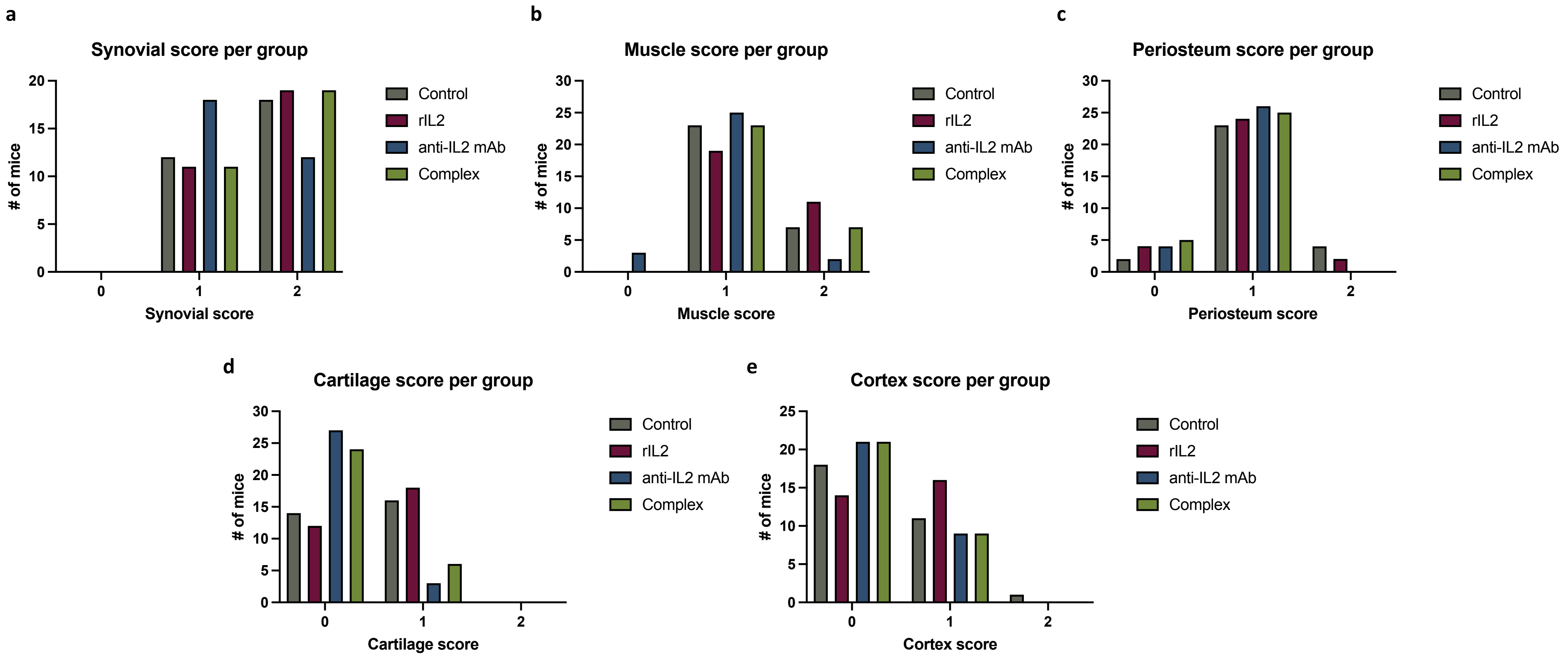

Supplemental Figure 1. Histological disease scores by component. Bars represent the number of mice per treatment group that received a score of 0, 1, or 2 for each histological component. Scores of 0 (no injury/inflammation) to 2 (significant injury/inflammation) were assessed for each histological component, including the synovium (A), skeletal muscle and soft tissue (B), periosteum (C), articular cartilage (D), and cortical bone (E).
